# A common variant in *CLDN14* causes precipitous, prelingual sensorineural hearing loss in multiple families due to founder effect

**DOI:** 10.1007/s00439-016-1746-7

**Published:** 2016-11-12

**Authors:** Justin A. Pater, Tammy Benteau, Anne Griffin, Cindy Penney, Susan G. Stanton, Sarah Predham, Bernadine Kielley, Jessica Squires, Jiayi Zhou, Quan Li, Nelly Abdelfatah, Darren D. O’Rielly, Terry-Lynn Young

**Affiliations:** 1 Craig L. Dobbin Genetics Research Centre, Discipline of Genetics, Faculty of Medicine, Memorial University, 300 Prince Phillip Drive, St. John’s, NL A1B 3V6 Canada; 2 Communication Sciences and Disorders, Western University, Elborn College, 1201 Western Road, London, ON N6G 1H1 Canada; 3 Department of Education and Early Childhood Development, Government of Newfoundland and Labrador, St. John’s, NL A1B 4J6 Canada; 4 Molecular Diagnostic Laboratory, Eastern Health, Craig L. Dobbin Genetics Research Centre, Faculty of Medicine, Memorial University, 300 Prince Phillip Drive, St. John’s, NL A1B 3V6 Canada

## Abstract

Genetic isolates provide unprecedented opportunities to identify pathogenic mutations and explore the full natural history of clinically heterogeneous phenotypes such as hearing loss. We noticed a unique audioprofile, characterized by prelingual and rapid deterioration of hearing thresholds at frequencies >0.5 kHz in several adults from unrelated families from the island population of Newfoundland. Targeted serial Sanger sequencing of probands for deafness alleles (*n* = 23) that we previously identified in this founder population was negative. Whole exome sequencing in four members of the largest family (R2010) identified a *CLDN14* (*DFNB29*) variant [c.488C>T; p. (Ala163Val)], likely pathogenic, sensorineural hearing loss, autosomal recessive. Although not associated with deafness or disease, *CLDN14* p.(Ala163Val) has been previously reported as a variant of uncertain significance (VUS). Targeted sequencing of 169 deafness probands identified one homozygote and one heterozygous carrier. Genealogical studies, cascade sequencing and haplotype analysis across four unrelated families showed all subjects with the unique audioprofile (*n* = 12) were also homozygous for p.(Ala163Val) and shared a 1.4 Mb *DFNB29*-associated haplotype on chromosome 21. Most significantly, sequencing 175 population controls revealed 1% of the population are heterozygous for *CLDN14* p.(Ala163Val), consistent with a major founder effect in Newfoundland. The youngest *CLDN14* [c.488C>T; p.(Ala163Val)] homozygote passed newborn screening and had normal hearing thresholds up to 3 years of age, which then deteriorated to a precipitous loss >1 kHz during the first decade. Our study suggests that genetic testing may be necessary to identify at-risk children in time to prevent speech, language and developmental delay.

**Electronic supplementary material:**

The online version of this article (doi:10.1007/s00439-016-1746-7) contains supplementary material, which is available to authorized users.

## Introduction

Hearing loss is one of the most common and genetic of all human phenotypes. Permanent bilateral sensorineural hearing loss affects 1/500 newborns, and almost twice as many adolescents (Smith et al. 1999; Morton and Nance 2006). Although approximately two-thirds of prelingual severe hearing loss cases are recessive, and 94 deafness loci have been reported, only a minority of hearing loss cases with a presumed recessive inheritance pattern can be conclusively diagnosed with a clear genetic etiology (Sloan-Heggen et al. 2016). Therefore, many recessive cases may be due to genetic defects in genes yet to be identified. However, recent studies using new high-throughput technologies and broader application in multi-ethnic populations report *GJB2* yields of less than 25%, suggesting a larger role for other recessive genes in prelingual severe cases (Yan et al. 2016; Sloan-Heggen et al. 2016).

Sensorineural hearing loss is characterized by both degree (mild, moderate, severe or profound) and configuration (low, mid and/or high frequency) using the standard behavioral audiogram. Although clinically heterogeneous, rare pathognomonic audiograms may present with surprising regularity in clinics within genetically isolated populations and where patients often share a common ancestor due to founder effects. For example, the Finnish and Pakistani populations have been invaluable for discovery of deafness genes as population bottlenecks (genetic drift) and/or inbreeding increase the likelihood of inheriting recessive alleles that are identical by descent. These populations are often characterized by large sibships, deep genealogies and higher consanguineous rates. The population of Newfoundland and Labrador (NL) was founded by ~20,000 Protestant English and Roman Catholic Irish settlers. Religious and geographic isolation within small coastal fishing (outport) communities (Manion 1977) has resulted in a higher inbreeding coefficient in the NL population (Bear et al. 1987, 1988; Zhai et al. 2015). We have previously identified several founder deafness mutations in the NL populations (Abdelfatah et al. 2013a, b; Ahmed et al. 2004; Doucette et al. 2009; Young et al. 2001).

A unique clinical audioprofile of steeply sloping sensorineural hearing loss was noted in several unrelated families. Herein, we report a founder missense variant in *CLDN14* causing precipitous prelingual sensorineural hearing loss in children born with normal hearing thresholds. The essential role of *CLDN14*, a component of tight junctions, was first discovered through studies in consanguineous families from the genetically isolated population of Pakistan. Tight junctions have been shown to play a significant role in maintaining the structural integrity of cells within the inner ear. Other genes encoding tight junction proteins, such as *MARVELD2* (*DFNB49*) (Riazuddin et al. 2006; Nayak et al. 2016), have also been implicated in recessive hearing loss. Claudin-14 is essential for the formation of tight junctions and is expressed in both hair cells and supporting cells of the organ of Corti; however, *CLDN14* exhibits preferential gene expression in sensory hair cells over supporting cells (Wilcox et al. 2001; Ben-Yosef et al. 2003; Scheffer et al. 2015). Initially, *CLDN14* was considered the cause of congenital recessive and profound deafness (Wilcox et al. 2001), and more recently of milder forms of hearing loss (Bashir et al. 2013). The *CLDN14* c. 488C>T p.(Ala163Val) allele has previously been reported in multiple studies as a variant of uncertain significance (VUS; Thorleifsson et al. 2009; Toka et al. 2013; Purcell et al. 2014) and recently identified by Sloan-Heggen et al. (2016) as one of two VUS in a patient with congenital hearing loss. Our study shows children inheriting two copies of *CLDN14* c. 488C>T p.(Ala163Val) alleles are born with normal hearing thresholds and experience a rapid and progressive loss by 3–4 years of age. Extensive clinical recruitment and targeted screening suggest that *CLDN14* p.(Ala163Val) represents a major founder variant in the Newfoundland population.

## Materials and methods

### Study participants and audiometric evaluations

This project is part of a large study of hereditary hearing loss in the Canadian province of Newfoundland and Labrador. Informed consent, family history and permission to access medical records and audiograms were obtained from all participants as per approved institutional review board protocol #01.186 (Human Research Ethics Board, St. John’s, NL, Canada). Sensorineural hearing loss was determined when hearing thresholds were abnormal, and the air and bone conduction results within 10 decibels (dB) of each other (i.e., air–bone gaps of 10 dB or less). Both retrospective and prospective audiograms and health records were obtained.

**Fig. 1:**
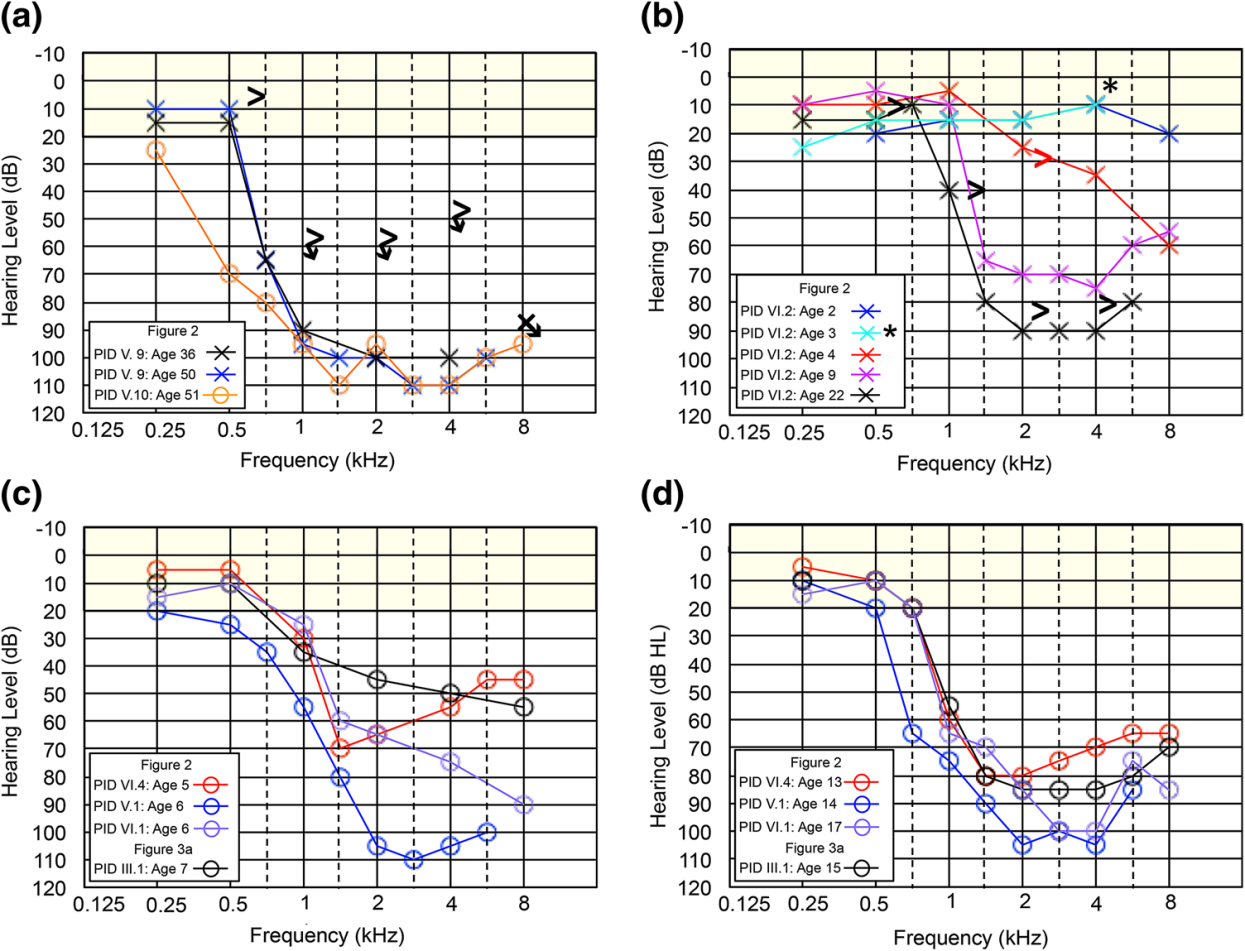
Rare, precipitous audiologic phenotype caused by *CLDN14* (c.488C>T; p.(Ala163Val)) in an Irish clan. **a** Pure tone audiogram of Family 2010 proband (PID V-9) and sister (PID V-10), **b** pure tone audiogram series for PID VI-2 (Family 2075) showing normal hearing at age 2 years and a progressive hearing loss apparent by 4 years of age, **c** first and **d** second decade pure tone audiogram of affected subjects. Yellow shaded area indicates range of normal hearing. Hearing thresholds are measured in decibels hearing level (dB HL), *X* = left ear (air conduction), *O* = right ear (air conduction), > = left ear (bone conduction), ↘ = no response at the limits of the audiometer. * = 8 kHz was not measured

**Fig. 2:**
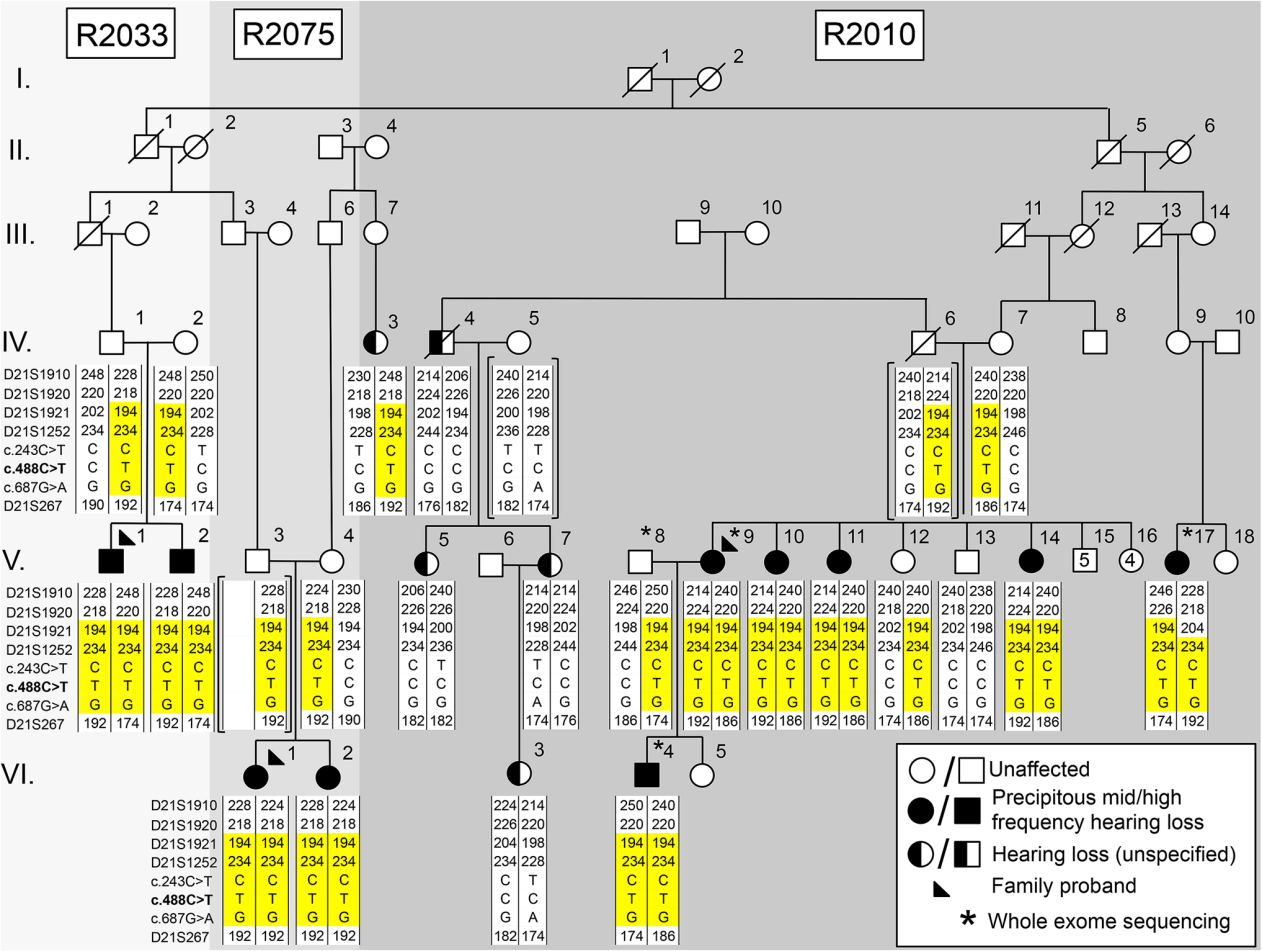
Combined pedigrees of three families (2033, 2075 and 2010) with rare, precipitous audiologic phenotype connect to a founding couple and share an ancestral *DFNB29*-associated haplotype.* Shaded symbols* precipitous sensorineural hearing loss.* Half-shaded symbols* unspecified hearing loss

In the course of ongoing clinical recruitment, we noted a rare but consistent clinical audioprofile characterized as steeply sloping, sensorineural hearing loss above 0.5 kHz with mid- and high-frequency thresholds in the severe to profound range (Fig. 1a–d). On the premise that subjects with this hearing loss pattern also shared a recent common ancestor, we used the distinct audioprofile to guide clinical recruitment, and a research team visited several small fishing villages (outports) to measure hearing thresholds, extend pedigrees, provide genetic counseling and collect blood samples (Fig. 2).

### DNA preparation, targeted sequencing and audioprofiling

Genomic DNA was extracted from peripheral blood using a simple salting out protocol (Miller et al. 1988). All probands recruited to the study were screened for population-specific deafness alleles (Supplementary Table 1; Abdelfatah et al. 2013a, b; Ahmed et al. 2004; Doucette et al. 2009; Young et al. 2001). To identify other candidate genes to screen, audiograms were submitted to Audiogene (Hildebrand et al. 2009) for computerized comparison with known average audiograms of 16 autosomal dominant loci (under the assumption that hearing loss was segregating as an autosomal dominant trait in these NL families). Bidirectional Sanger sequencing (ABI PRISM 3500XL DNA Analyzer; Applied Biosystems, Foster City, CA, USA) with standard PCR assay using Primer3 (Untergasser et al. 2012) was used to screen candidate mutations and genes (Merner et al. 2008). We used Mutation Surveyor Software (version 4.07, SoftGenetics LLC State College, PA 16803) to select quality reads and analyze DNA sequences.

### Whole exome sequencing and variant filtration

We prepared whole exome libraries for four members of Family 2010 using the Ion Torrent AmpliSeq RDY Exome Kit (Life Technologies, Cat. #A27193) (Fig. 2). Exome library purification, adapter ligation and barcoding were done using the Ion PI Hi-Q OT2 200 kit (Life Technologies, Cat. #A26434). Purified libraries were quantified using the Ion Library Quantification Kit (Life Technologies, Cat. #4468802) and then loaded onto a PI v3 chip and sequenced with the Ion Torrent Proton Sequencer. Single-nucleotide variants (SNVs) and insertion/deletions (INDELs) were called (GATK, v3.5) and annotated using SnpEff (v4.1; http://snpeff.sourceforge.net/) and SNVs were filtered against publically available SNP databases (ExAC Browser, http://exac.broadinstitute.org/; dbSNP, http://www.ncbi.nlm.nih.gov/projects/SNP/; 1000 genomes, http://www.1000genomes.org). We assessed the impact of SNVs at the protein level with SIFT, PolyPhen-2, and MutationTaster. Filtered SNVs had a minimum of 20× coverage, a predicted moderate/high impact (nonsense, frameshift, missense, splice sites) and a minor allele frequency (MAF) of <1%. The apparent vertical transmission of hearing loss in several branches of the clan pedigree (Fig. 2) could be due to either a dominant gene with reduced penetrance, or a recessive gene with a pseudodominant inheritance pattern; therefore, we conducted both autosomal dominant and recessive analyses.

### Cascade sequencing and haplotype analysis

Potential pathogenic mutations were subjected to cascade screening in all available relatives across three families observed to have the same rare audioprofile (Families 2010, 2033 and 2075) and also in 175 ethnically matched controls. Microsatellites flanking candidate genes were genotyped according to standard procedures (Abdelfatah et al. 2013a) and alleles called using GeneMapper software v4.0. Haplotypes were reconstructed manually and compared across families. Variants of interest were also screened in 169 deafness probands with Newfoundland ancestry.

## Results

### Clinical evaluation

Our research audiologist (AG) noted that probands (from Families 2010, 2075 and 2033) all shared a unique hearing loss pattern. The proband of Family 2010 (V-9; Fig. 2) presented at 36 years of age with the characteristic pattern of normal low-frequency thresholds, steeply sloping to severe bilateral, symmetrical, sensorineural hearing loss throughout mid and high frequencies (Fig. 1a). Age appropriate audiologic tests of the proband’s son (VI-4; Fig. 2) at 1 month and 1 year of age were normal. Serial audiograms on PID VI-2 (Fig. 1b; Family 2075) show normal hearing thresholds across frequencies up to 3 years of age, and subsequent rapid progression of hearing loss affecting high frequencies first. Significant hearing loss of variable severity is already present in children aged 5–7 years (Fig. 1c), which include probands of families 2033 and 2075. By the middle of the second decade of life, these children uniformly exhibit the distinctive steeply sloping audiogram (Fig. 1d). The hearing loss progresses slowly during subsequent decades, primarily in the mid–high frequencies, with relatively well-preserved low-frequency thresholds. For adults, some variation in thresholds at 0.5 kHz is observed (PID V-10; Fig. 1a) but otherwise the adult presentation is relatively uniform.

### Targeted sequencing and audioprofiling

Targeted sequencing was carried out on probands for known deafness alleles (previously identified in this population; Supplementary Table 1) but none were found. Several gene candidates, as suggested by Audiogene (Hildebrand et al. 2009), were also Sanger sequenced, including *COCH*, *KCNQ4* and *TMC1*. We identified a rare variant in *TMC1* (c.421C>T; MAF of 0.01%) predicted to cause the substitution of arginine to a tryptophan residue at position 141 and to be deleterious by SIFT (and probably damaging by PolyPhen-2 and Panther). Although identified in both the probands (V-11) of Family 2010 and transmitted to her affected son (V-14), the c.421C>T variant did not co-segregate with mid–high-frequency loss in this family (data not shown).

### Whole exome sequencing

**Fig. 3:**
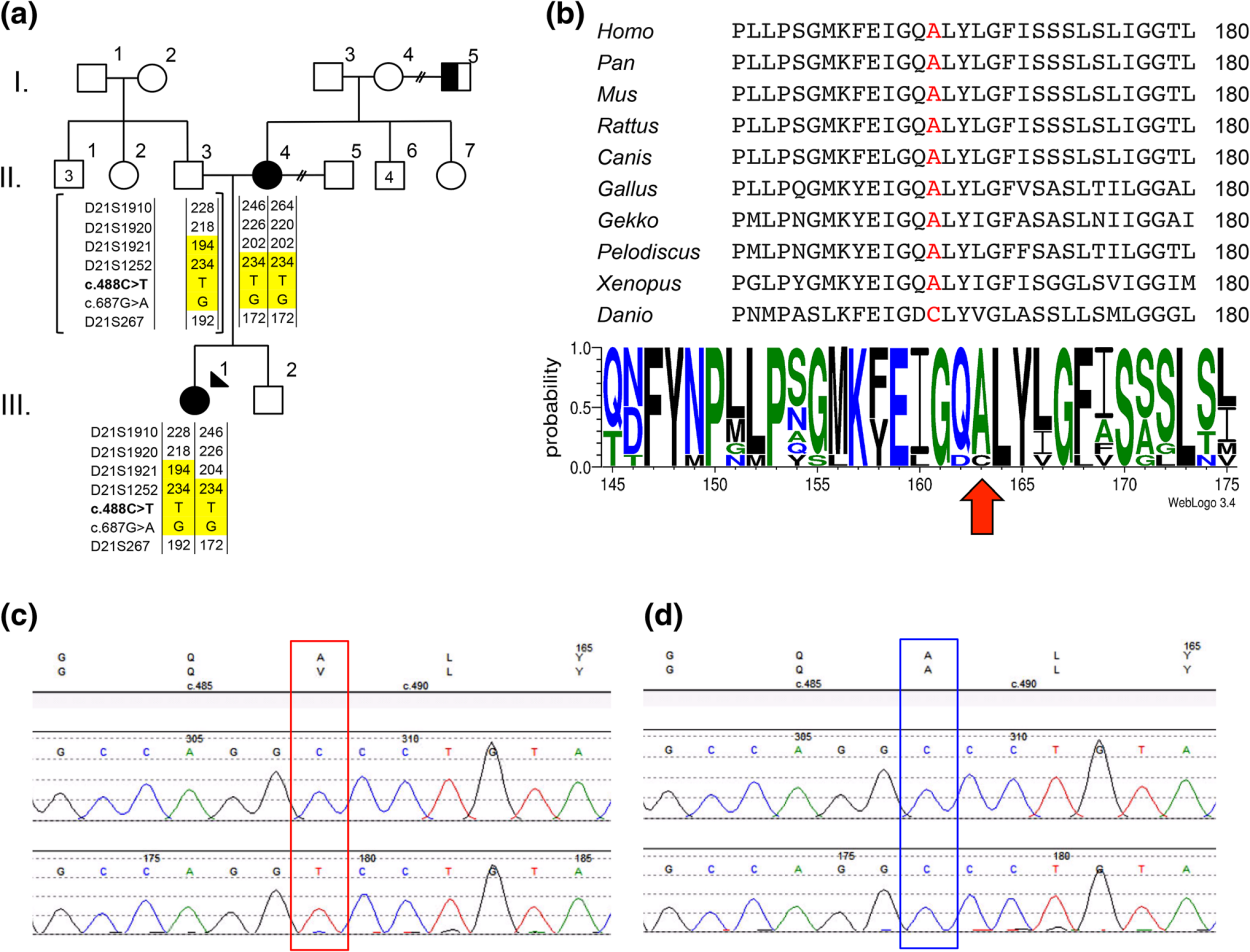
**a** Pedigree of family R2072, identified in screening of the NL deafness cohort, with the rare, precipitous audiologic phenotype who also share the *CLDN14* [c.488C>T; p.(Ala163Val)] variant and ancestral *DFNB29*-associated haplotype, **b** Conservation of the Claudin-14 protein using Clustal Omega and WebLogo display. *Homo sapiens* (NP_001139551.1), *Pan paniscus* (XP_008975916.1), *Mus musculus* (NP_001159398.1), *Rattus norvegicus* (NP_001013447.1), *Canis lupus familiaris* (XP_013965166.1), *Gallus gallus* (XP_015155717.1), *Gekko japonicas* (XP_015277878.1), *Pelodiscus sinensis* (XP_006126056.1), *Xenopus laevis* (NP_001086045.1), *Danio rerio* (NP_001004559.2).* Red font and arrow* indicate a highly conserved alanine residue at position 163, **c** sequence electropherograms of *CLDN14* [c.488C>T; p.(Ala163Val)]. *Box* highlights variant, **d** wild-type/normal *CLDN14* c.488C. *Box* highlights normal sequence

**Fig. 4:**
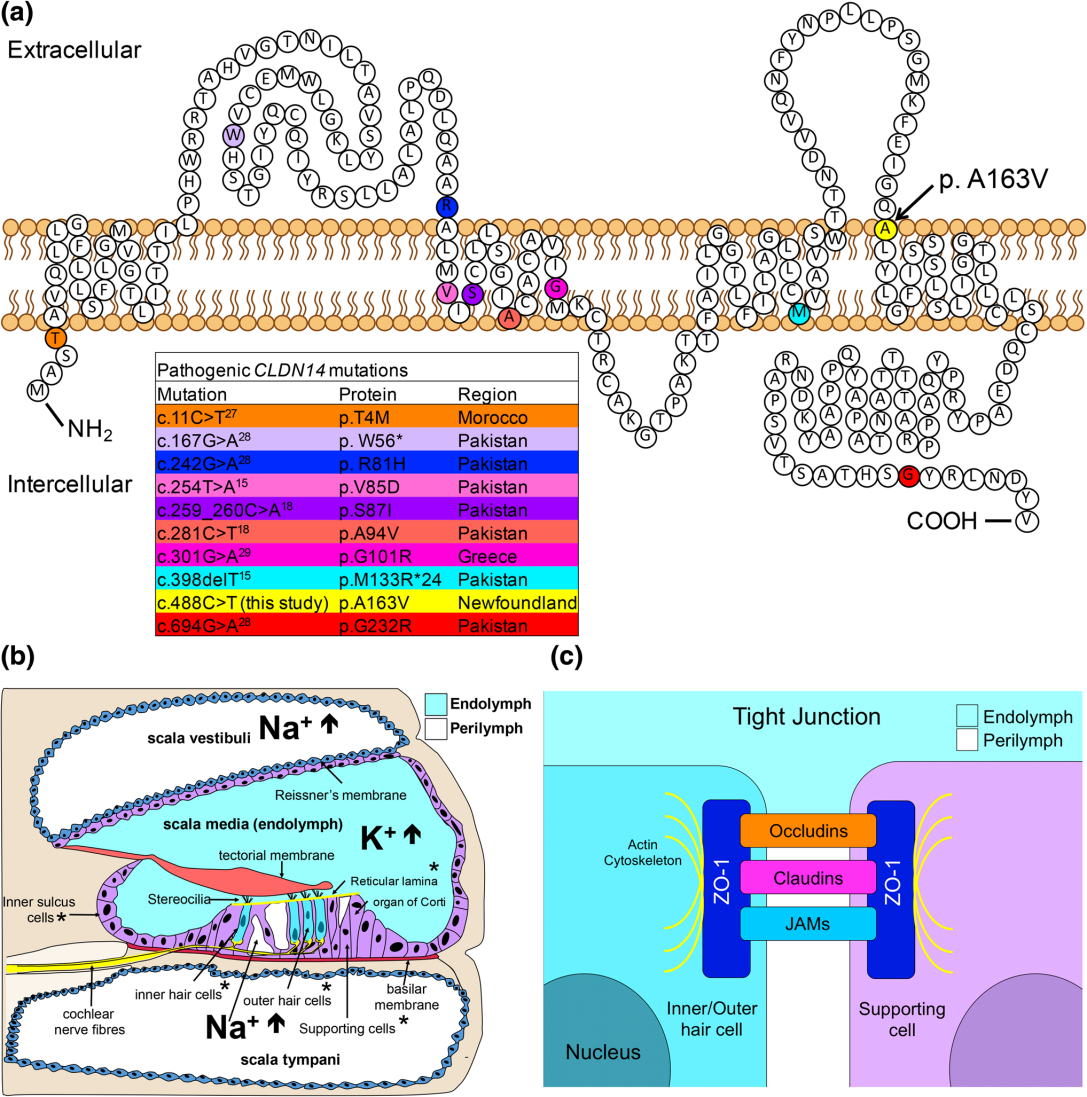
**a** Location of pathogenic mutations in Claudin-14. Colored amino acid residues indicate previously reported claudin-14 mutations.* Arrow* indicates position of *CLDN14* c.488C>T [p.(Ala163Val)]. Adapted from: Bashir et al. (2013), **b** Cross-sectional diagram illustrating the anatomical location of the cochlear canals and their respective ionic composition. *CLDN14 expression, **c** Schematic diagram demonstrating the molecular structure of tight junctions

Whole exome sequencing on Family 2010 using three affecteds (V-9, VI-4 and V-17) and one unaffected parent (V-8) (Fig. 2) yielded >35,000 total variants. Under a dominant model, 34 heterozygous variants were filtered (data not shown). However, none of these variants resided within known deafness genes/loci (http://hereditaryhearingloss.org/). Under a recessive model, we filtered four homozygous variants (in *PRKDC*, *ZNF404*, *CUL7* and *CLDN14*). One of these, *CLDN14* (*DFNB29*) is a known deafness gene expressed in the sensory epithelium of the organ of Corti of the inner ear (Wilcox et al. 2001; Scheffer et al. 2015). *CLDN14* consists of three exons and two isoforms and encodes a protein containing four transmembrane domains. The *CLDN14* p.(Ala163Val) point variant (Fig. 3c) identified in Family 2010 predicts substitution of an alanine to a valine at the beginning of the fourth transmembrane domain (Fig. 4a) and is highly conserved (Fig. 3b). The *CLDN14* c.488C>T allele was first identified in the Icelandic population (Thorleifsson et al. 2009). Globally, the *CLDN14* c.488C>T variant has an MAF of 0.02564% (ExAC Browser, http://exac.broadinstitute.org/) and has been reported in both European and African populations. The heterozygous *CLDN14* (human GRCh37/hg19: g. 37833506 G>A, NM_012130.3: c.488 C>T) allele is reported as a variant of uncertain significance in dbSNP (rs143797113), ExAC browser (MAF: 0.02564%), 1000 genomes (MAF: 0.04%), and the Grand Opportunity Exome Sequencing Project (MAF: 0.05%). This allele has also been reported in several control samples from other study cohorts within the USA (Toka et al. 2013), Sweden (Purcell et al. 2014), and Africa (ExAC browser). The majority of known pathogenic *CLDN14* mutations reside within one of the transmembrane domains in Claudin-14 (Fig. 4a; Bashir et al. 2013; Charif et al. 2013; Lee et al. 2012; Wattenhofer et al. 2005; Wilcox et al. 2001). Functional studies of *CLDN14* mutations have demonstrated the importance of transmembrane domains with respect to protein topology and folding, as well as proper spatial localization within cells. For example, previous localization experiments showed that the p.V85D and p. G101R deafness mutations within domain II (Fig. 4a) fail to form tight junctions due to the mislocalization of Claudin-14 protein to the cytoplasm, in vitro (Wattenhofer et al. 2005). Since p. (Ala163Val) is predicted to change a highly conserved amino acid within the fourth transmembrane domain (Fig. 3b), we suspect a similar impact regarding the spatial localization of claudin-14 to the plasma membrane, leading to the cells’ inability to form tight junctions. While previous research have demonstrated the importance of amino acid conservation within in claudin-14 transmembrane domains, experimental functional studies are warranted to prove *CLDN14* c.488C>T, p.(Ala163Val) pathogenicity.

### Cascade sequencing and haplotype analysis

Cascade sequencing in all available subjects from Families 2033 and 2075 show that affecteds with the distinct precipitous mid–high-frequency hearing loss (Fig. 2, filled symbols) were also homozygous for *CLDN14* p.(Ala163Val) (Fig. 3c). Subjects with a flat loss, such as PID IV-4 and his descendants (V-5, V-7 and VI-4) lacked the recessive *CLDN14* variant (Fig. 3d). This pattern is consistent with our hypothesis that *CLDN14* p.(Ala163Val) is a likely pathogenic, recessive allele where homozygosity results in a distinct precipitous mid–high-frequency hearing loss and relatives inheriting a single copy (carriers) or wild type do not have this pattern. According to the American College of Medical Genetics standards and guideline (Richards et al. 2015), *CLDN14* p.(Ala163Val) is a strong PS4 PM2 likely pathogenic variant. The cause of hearing loss in subjects with flat audioprofiles is not known, but is clearly not due to homozygosity for *CLDN14* c.488C>T. Future studies will explore the genetic etiology of their hearing loss. Furthermore, screening our cohort of 169 deafness probands identified an additional homozygous subject (Family 2072) and two heterozygous carriers. In Family 2072, the proband’s mother (with the distinct audioprofile) was also found to be homozygous for *CLDN14* p.(Ala163Val) and her father a carrier (Fig. 3a). Screening population controls identified four carriers out of 175 subjects, estimating an MAF of 1.15% in the Newfoundland population and suggesting that this likely pathogenic variant is not rare.

Extensive genotyping in the vicinity of *DFNB29* revealed that p.(Ala163Val) resides on a 1.4 Mb ancestral haplotype shared across all four families (Figs. 2, 3a). Haplotype analysis shows affected individuals in the four families inherit an ancestral *DFNB29*-associated haplotype on chromosome 21q22.1, signifying clan membership, although biological connection for Family 2072 was not found (Fig. 3a). Additionally, we sequenced all coding sequences of the *CLDN14* gene, including the exon/intron boundaries and 5′ and 3′ UTRs. We identified a common synonymous variant (c.243C>T; rs219799) within the clan, which was incorporated into our DFNB29-associated deafness haplotype.

### Genealogical analysis

**Fig. 5:**
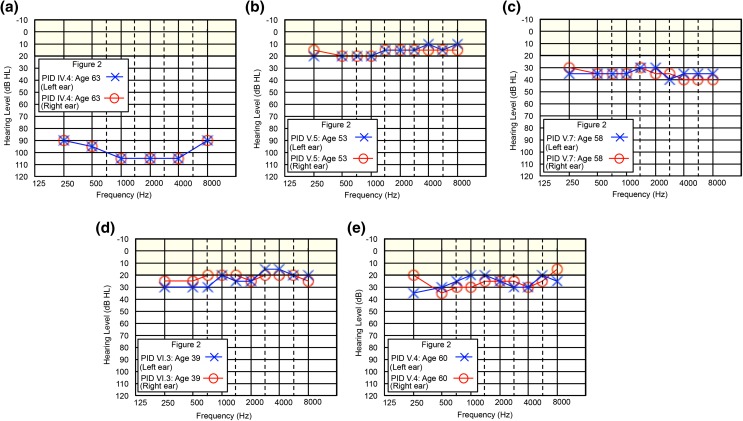
Clan members lacking the recessive *CLDN14* [c.488C>T; p.(Ala163Val)] variant do not present with the characteristic steeply sloping hearing phenotype, exhibiting a different age of onset and hearing threshold progression **a** Profound, flat sensorineural hearing loss with an unknown etiology at age 63 (PID IV-4), **b** PID V-5 (age: 53) presents with borderline hearing thresholds, **c** PID V-7 (age: 58) presents with mild hearing loss with a diagnosis of Meniere’s disease, **d** at age 39, PID VI-3 presents with mild hearing loss, and **e** a heterozygous *CLDN14* [c.488C>T; p.(Ala163Val)] carrier (PID V-4) exhibits mild hearing loss at age 60. *Yellow shaded area* indicates range of normal hearing. Hearing thresholds are measured in decibels hearing level (dB HL), X = left ear (air conduction), O = right ear (air conduction)

Extension of the pedigrees and review of all clinical audiograms identified 16 subjects with hearing loss; 10/16 subjects showed the distinct precipitous mid–high-frequency hearing loss (Fig. 2). Subjects with hearing impairment not consistent with the distinct precipitous mid–high-frequency pattern include PID IV-3 (whom we have not connected to the founding couple) and all descendants of PID IV-4 (Fig. 2). In these cases, the audiogram can be characterized as a flat loss across all frequencies: PID IV-4 had a profound flat loss and his descendants (V-5, V-7, VI-3) show a mild flat loss (Fig. 5). Family interviews determined the surnames suggestive of Irish descent (Seary 1977) and connected Families 2010, 2033 and 2075 to a single founding couple six generations ago. We noted that the inheritance pattern in the combined pedigree suggested either autosomal dominant (with reduced penetrance) or autosomal recessive (pseudodominant) inheritance (Fig. 2). In summary, this population-based study using a targeted and whole exome sequencing approach identified a *CLDN14* (*DFNB29*) variant (c.488C>T, p. (Ala163Val), likely pathogenic, sensorineural hearing loss, autosomal recessive.

## Discussion

We have determined that a known VUS (*CLDN14*, c.488C>T, p. (Ala163Val)) is likely pathogenic, and causes a precipitous, bilateral and rapid deterioration of hearing thresholds at frequencies >0.75 kHz in children, progressing gradually in adults. We have also determined that this likely pathogenic variant is amplified in the founder population of the island of Newfoundland and is present in ~1% of the population. The role of *CLDN14* in nonsyndromic hearing loss was first described in two large consanguineous families from Pakistan with recessive profound congenital deafness (Wilcox et al. 2001). Recessive *CLDN14* alleles manifest as nonsyndromic sensorineural hearing loss with considerable phenotypic variability and may present as congenital or prelingual, and mild, moderate–severe or profound (Bashir et al. 2010, 2013). In this study, homozygous children had normal hearing thresholds up to 3 years of age and overall, a remarkably conserved hearing phenotype. A combination of pedigree extension and genotyping linked four families of Irish ancestry to a founding couple six generations back.

The claudin family of proteins consists of 24 members with tissue-specific expression. Claudin-14 plays a critical role in the formation of tight junction barriers that regulate paracellular ion transport (Mineta et al. 2011) and is highly expressed in the kidney, liver and the inner ear (Ben-Yosef et al. 2003; Wilcox et al. 2001). Moreover, preferential gene expression has been observed in the inner ear, as *CLDN14* expression is lower in supporting cells, relative to sensory hair cells (Wilcox et al. 2001; Scheffer et al. 2015). Normal hearing function and hair cell depolarization are dependent on tight junctions in the reticular lamina. In the organ of Corti, hair cell stereocilia are bathed in potassium-rich endolymph, while the basolateral surface of the hair cell body is surrounded by an intercellular (or extracellular) fluid continuous with the perilymph (Fig. 4b, c). The reticular lamina, formed in part by tight junctions between the apical surfaces of hair cells and supporting cells of the sensory epithelium, creates a barrier isolating the endolymphatic fluid from other cochlear compartments, which contain perilymph. Maintenance of this ionic gradient is essential for mechanotransduction, which depends on the modulation of potassium current flowing from the endolymph into the hair cells through the stereocilia as they are displaced by sound-induced vibrations. Disruption of this tight junction barrier alters the ionic gradient, increasing the potassium concentration around the hair cell body, compromising mechanotransduction and causing hair cell toxicity and eventual cell death.

The *CLDN14* p.(Ala163Val) variant reported in this study has been identified in previous studies but not in association with disease. It was first reported as a VUS by Thorleifsson et al. (2009) in a large Iceland/Netherlands GWAS cohort study examining SNPs associated with kidney stones and bone mineral density and more recently by Toka et al. (2013), who detected the *CLDN14* p.(Ala163Val) allele in 3 of 1230 study participants for another kidney function study. The heterozygous p.(Ala163Val) allele was also found in a Swedish GWAS study examining the polygenic nature of schizophrenia (Purcell et al. 2014). The heterozygous p.(Ala163Val) allele was submitted 31 times to ExAC browser, 29 alleles from European descent and 2 from the African population. In a recent American study including 1119 deafness probands, a cohort made up of 62.3% autosomal recessive cases (Sloan-Heggen et al. 2016) used a targeted sequencing approach and the most commonly implicated genes were *GJB2*, *MYH9*, *OTOA*, *PCDH15*, *SLC26A4*, *STRC*, *TMC1*, *TMPRSS3* and *USH2A*. Interestingly, Sloan-Heggen et al. 2016 reported the p.(Ala163Val) allele in a patient with congenital hearing loss; however, in a compound heterozygous state with a second *CLDN14* allele (p.P28L). In summary, these studies suggest that the likely pathogenic *CLDN14* p.(Ala163Val) allele is both rare and widely distributed around the globe.

Many reports claim that approximately 50% of autosomal recessive deafness is caused by either homozygous or compound heterozygous mutations in the *DFNB1* locus (*GJB2*), which is often the only gene that is routinely screened in the clinical setting when there is a family history of hearing loss. This represents a massive ascertainment bias, as children who are *DFNB1* negative are not followed up, due to the expenses associated with genetic testing. Recently, a large, ethnically diverse, cohort study demonstrated the importance of investigating *DFNB1*-negative deaf probands (Yan et al. 2016). This study took a targeted panel approach in 342 probands (185 simplex and 157 multiplex families), sequenced 180 known hearing loss genes, and identified 151 variants in 119 families. Fifty-three families had pathogenic or likely pathogenic mutations within 27 genes, while the remaining were variants of uncertain significance. This study solved 25 and 7% of multiplex and simplex families, respectively, emphasizing the importance of large families and strong histories of disease in genetic studies.

Pediatric hearing programs strive to identify and treat early to prevent delay in language, learning and social development. However, the detection of delayed onset and progressive forms of hearing loss remain a significant challenge. Children who are homozygous *CLDN14* p.(Ala163Val) pass newborn and early hearing tests. The proband’s son (R2010, PID VI-4) was discharged after his test at 1 year of age indicated normal hearing. Preschool testing 4 years later showed significant deterioration of both mid and high frequencies (Fig. 1c). Delayed identification could result from limited testing of high frequencies in the preschool years, often complicated by limited testing tolerance in children. In this study, PID VI-2 had normal hearing at 8 kHz at 2 years of age. At 3 years, hearing was reported normal although thresholds at 8 kHz were not performed. By 4 years, a 55 dBHL threshold at 8 kHz and mild to moderate loss at all high frequencies required immediate hearing aid fitting. Retrospectively, if 8 kHz thresholds had been performed at 3 years, diagnosis and therapy could have been offered a year earlier (Fig. 1b). Conversely, genetic testing or prenatal/preconception parental carrier screening could provide appropriate hearing surveillance and minimize the risk of delays in language development and learning from rapidly progressing hearing loss.

Adults who are homozygous for *CLDN14* p.(Ala163Val) also have a consistent phenotype but challenges in management remain. Hearing aids benefit affected children and young adults (up to the third decade), but most adults do not find them beneficial. For example, PIDs V-9 (age: 50), V-10 (age: 51) and V-17 (age: 57; Fig. 2) reported some additional sound with hearing aids but no improvement of speech comprehension, consistent with the extreme erosion of mid and high frequencies. Older affected adults with well-preserved low-frequency sensitivity have limited communication by phone. PIDs V-10 and V-17 (Fig. 2) whose threshold at 0.5 kHz is deteriorated can no longer communicate by phone. Adult members of this clan are highly skilled speech readers who can detect speech initiation and turn quickly to maximize the use of visual clues. Unfortunately, these skills can be mistaken for hearing and subjects have voiced concerns regarding safety in the workplace.

The development of the organ of Corti is unidirectional, and follows a base-to-apex hair cell degeneration in the *Cldn14*-null mouse cochlea. This may explain why we observe a sensorineural threshold loss progressing from the high to low frequencies in affected clan members. The cochlea discerns high- from low-frequency sound, based on a stiffness gradient along the basilar membrane (Ehret 1978; Teudt and Richter 2014). In *Cldn14*-null mice, the organ of Corti undergoes a base-to-apex deterioration beginning around postnatal day 10, with a more severe and rapid degeneration of outer hair cells compared to inner hair cells. By day 13, the three rows of outer hair cells are almost completely absent in the cochlear base, with partial loss and stereociliar disorganization in the middle and apical turns (Ben-Yosef et al. 2003). The cochlear lesion then proceeds towards the cochlear apex, with a rapid deterioration of the outer hair cells accompanied by the onset of inner hair cell damage. By day 18, outer hair cell deterioration is severe with only a few remaining outer hair cells exhibiting damaged stereocilia in the most apical region; in contrast, only partial inner hair cell loss is reported throughout the cochlea by this age. Auditory brainstem responses measured in 4-week-old *Cldn14*-null mice indicate a significant hearing loss in comparison to their wild-type and heterozygous littermates (Ben-Yosef et al. 2003).

## Summary

A population-based study of hearing loss in the NL population has clarified the role of *CLDN14* p.(Ala163Val), a VUS previously identified in the USA, Iceland and Sweden. *CLDN14* p.(Ala163Val) appears to be of Irish origin and causes a precipitous, prelingual recessive sensorineural hearing loss. This likely pathogenic variant is frequent in this island population of Northern European decent, and *CLDN14* p.(Ala163Val) homozygotes have normal hearing thresholds at birth, then experience rapid, progressive nonsyndromic hearing loss in early childhood. Although missed by newborn hearing screening, genetic testing would ensure identification of at-risk children, allowing for appropriate monitoring and timely intervention, aural rehabilitation and counseling for families. Although *GJB2* is routinely screened in the regional hospital diagnostic clinic, we recommend targeted screening of *CLDN14*, as well.

### Limitations

While exome sequencing is a powerful tool to elucidate disease causing, coding variant, it does not explore non-coding regions. Additionally, there is no experimental proof of the predicted amino acid substitution, and without functional data, we cannot be certain that the point mutation impacts protein location within tight junctions. For example, this variant could cause alternative splicing or alter gene expression. Although less likely, it is plausible that a causal, non-coding variant at the *DFNB29* locus is in linkage disequilibrium with p.(Ala163Val). Even though our study presents several lines of evidence to suggest pathogenicity, experimental functional analysis of p.(Ala163Val) is required.

## Electronic supplementary material

Below is the link to the electronic supplementary material.

Supplementary material 1 (PDF 56 kb)
