# Supplementary material for: A common variant in CLDN14 causes precipitous, prelingual sensorineural hearing loss in multiple families due to founder effect
Source: Hum Genet. 2016 Nov 12;136(1):107–18. doi: 10.1007/s00439-016-1746-7 (PMC5215284; doi:10.1007/s00439-016-1746-7)
Supplement: Supplementary file 1 — Supplementary material 1 (PDF 56 kb) [file 439_2016_1746_MOESM1_ESM.pdf]

Article Title: A common mutation in *CLDN14* causes precipitous, prelingual sensorineural hearing loss in multiple families due to founder effect
Journal name: Human Genetics

Author names: Justin Pater, Tammy Benteau, Anne Griffin, Cindy Penney, Susan G. Stanton, Sarah Predham, Bernadine Kielley, Jessica Squires, Jiayi Zhou, Quan Li, Tammy Benteau, Nelly Abdelfatah, Darren D. O'Rielly, Terry-Lynn Young
Affiliation: Craig L. Dobbin Genetics Research Centre, Faculty of Medicine, Memorial University, 300 Prince Phillip Drive, St. John’s, Newfoundland and Labrador, Canada, A1B 3V6
Email address: tlyoung@mun.ca

Supplementary Table 1. Recurrent Hearing Loss Mutations in Newfoundland

Gene	Accession No.	Variant	DFN Locus	Locus (Cyto)
TMPRSS3	NM_024022	c. 207delC	DFNB8/10	21q22
		c. 268 G>C		
		c. 782+3delGAG		
		c. 757 A>G		
		c. 612-2insTA		
WFS1	NM_006005.3	c. 2146 G>A	DFNA6/38	4p16.3
		c. 1832 A>G		
PCDH15	NM_033056	c. 1583 T>A	DFNB23	10p11.2-q21
		c. 1590 + 20 A>G		
KCNQ4	NM_004700	c. 806delCCT	DFNA2A	1p34
GJB2	NM_004004	c.-23+1G>A	DFNA3A/B1	13q11-q12
		c. 35delG		
		c. 101 T>C		
		c. 229 T>C		
		c. 249 C>G		
		c. 167delT		
GJB6	NM_006783	delD1351830	DFNA3B	13q12
		delD1351854		
GJB3	NM_024009.2	c. 109 G>A	DFNA2B	1p35.1
SMPX	NM_014332.2	c. 99delC	DFNX4	Xp22
COCH	NM_004086.2	c. 151 C>T	DFNA9	14q12-q13
TECTA	NM_005422	c. 26557 A>G	DFNB21	11q
TMC1	NM_138691	c. 421C>T	DFNA36	9q13-a21
